# Evaluating the Psychometric Quality of Social Skills Measures: A Systematic Review

**DOI:** 10.1371/journal.pone.0132299

**Published:** 2015-07-07

**Authors:** Reinie Cordier, Renée Speyer, Yu-Wei Chen, Sarah Wilkes-Gillan, Ted Brown, Helen Bourke-Taylor, Kenji Doma, Anthony Leicht

**Affiliations:** 1 School of Occupational Therapy and Social Work, Curtin University, Perth, WA, Australia; 2 College of Healthcare Sciences, James Cook University, Townsville, QLD, Australia; 3 Faculty of Health Sciences, The University of Sydney, Sydney, NSW, Australia; 4 School of Allied Health, Australian Catholic University, North Sydney, NSW, Australia; 5 Department of Occupational Therapy, Faculty of Medicine, Nursing and Health Sciences, Monash University–Peninsula Campus, Frankston, VIC, Australia; 6 School of Allied Health, Australian Catholic University, St Patricks Campus, Fitzroy, VIC, Australia; University of New South Wales, AUSTRALIA

## Abstract

**Introduction:**

Impairments in social functioning are associated with an array of adverse outcomes. Social skills measures are commonly used by health professionals to assess and plan the treatment of social skills difficulties. There is a need to comprehensively evaluate the quality of psychometric properties reported across these measures to guide assessment and treatment planning.

**Objective:**

To conduct a systematic review of the literature on the psychometric properties of social skills and behaviours measures for both children and adults.

**Methods:**

A systematic search was performed using four electronic databases: CINAHL, PsycINFO, Embase and Pubmed; the Health and Psychosocial Instruments database; and grey literature using PsycExtra and Google Scholar. The psychometric properties of the social skills measures were evaluated against the COSMIN taxonomy of measurement properties using pre-set psychometric criteria.

**Results:**

Thirty-Six studies and nine manuals were included to assess the psychometric properties of thirteen social skills measures that met the inclusion criteria. Most measures obtained excellent overall methodological quality scores for internal consistency and reliability. However, eight measures did not report measurement error, nine measures did not report cross-cultural validity and eleven measures did not report criterion validity.

**Conclusions:**

The overall quality of the psychometric properties of most measures was satisfactory. The SSBS-2, HCSBS and PKBS-2 were the three measures with the most robust evidence of sound psychometric quality in at least seven of the eight psychometric properties that were appraised. A universal working definition of social functioning as an overarching construct is recommended. There is a need for ongoing research in the area of the psychometric properties of social skills and behaviours instruments.

## Introduction

### Social Functioning

Most theorists agree that social functioning is a complex construct that encompasses social skills as well as social behaviour and cognition during inter-personal interactions [1]. Social functioning involves the integration of emotional, linguistic and cognitive skills, which develop from early childhood to adolescence [1]. Social functioning is foundational for the development and maintenance of meaningful relationships and community participation and is critical for both physical health and psychological well-being [2]. Impairments in social functioning manifest in approximately one in every ten children [3], with greater levels of social impairment reported in developmental disabilities [4–9]. Impairments in social functioning are associated with an array of adverse outcomes in adolescence and adulthood, such as delinquency [10], social withdrawal and isolation [11].

### Theoretical Frameworks on Social Functioning

Theoretical models of social functioning are most commonly embedded within psychology; with more recent models emerging from neuroscience [1]. These models are commonly embedded within a social information processing (SIP) framework and are focused on infant development and the cognitive processes needed for social skills in adults [12]. Researchers have extended SIP to include both cognitive and affective dimensions. In their extensions of SIP, Mostow et al. [13] and Guralnick [14] included emotional, cognitive and behavioural predictors of peer related social competence.

While few comprehensive theoretical models exist, analogous perspectives and definitions are evident throughout literature [1, 15–18]. There appears to be consensus among theorists that social functioning is an overarching construct which is reliant on a range of cognitive, emotional and linguistic skills; reflecting a person’s overall performance in the area of social development [1, 19, 20].

#### Cognitive functions, social emotional and linguistic skills

In their model of socio-cognitive integration of abilities, Beauchamp and Anderson [1] considered social skills and functions from a range of perspectives, integrating them into a model of social competence. The socio-cognitive integration of abilities model defines the core dimensions of social functioning (biological–psychological–social) and their interactions within a developmental framework founded on empirical research and clinical principles.

Cognitive and executive functions have been central to most models of social functioning [1]. In most models, cognitive function is used to reflect a range of higher cognitive processes, such as: attentional control (e.g., selective and sustained attention, response inhibition, self-monitoring, self-regulation) and skills linked to executive functioning (e.g., working memory, planning, problem-solving, strategic behaviour). Researchers have linked deficits in these skills to poor social outcomes, including: antisocial behaviour, emotional dysregulation, delinquency and peer-rejection [21–23].

Another principal component of most social functioning models is socio-emotional skills. These skills have been reported to include: face-emotion perception, theory of mind, and empathy. Face-emotion perception is fundamental to recognising emotion, which is needed for reciprocal social interactions [24, 25]. Theory of mind is a social cognitive skill and involves understanding the emotion, intention and perceptions of others and how the knowledge or beliefs of someone else may differ from one’s own [26]. Empathy involves identifying the emotional state of another, the capacity to take the perspective or role of the other, and the evocation of shared affective responses. Empathy is associated with pro-social behaviour and comprises both affective and cognitive components [27–29].

There is an array of literature highlighting the influence of communication skills on social functioning. However, linguistic skills are infrequently incorporated into models of social functioning [1, 30]. In particular, pragmatic language skills have been described as fundamental to social functioning as they are needed to: a) integrate verbal and non-verbal communication; b) detect and interpret underlying meaning in social cues; c) respond appropriately during social interactions; and d) regulate emotions [30–32].

#### Approaches to social development and social competence

Some theorists view social functioning from a hierarchical perspective with ‘social skills,’ and ‘social cognition’ representing different levels of social behaviour under the auspice of social functioning. However, other theorists use the constructs ‘social competence’, ‘social skills’ and ‘social functioning’ interchangeably. In developmental literature, there is general consensus that children may exhibit externalising or internalising behaviour when social competence is lacking [33]. Social competence has often been conceptualised to reflect effectiveness or success in social interactions. However, there is a noteworthy lack of agreement on the nature of its relationship to social functioning (if viewed as a separate construct) and how to define, measure and approach the skills attributable to social competence [33].

Researchers have adopted four approaches to social functioning and competence in the field of social development: 1) a social skills approach, focusing on specific skills and pro-social behaviours; 2) a peer status approach, focusing on sociometric status and peer acceptance or rejection; 3) a relationship approach, based on one’s ability to form and maintain friendships and positive relationships with parents, teachers and intimate relationships in adulthood; and 4) an adaptive approach, that takes into account the need for individuals to adjust their social interactions and behaviour across a variety of contexts and types of social situations [16, 34]. Despite these divergent approaches to social functioning, there is consensus among theorists regarding social functioning. There is also agreement among theorists that these skills and behaviours are mediated by a range of internal and external factors.

#### Mediating factors of social development and social functioning

Much research has focused on factors that mediate social functioning. Mediating factors have often been categorised as external factors or internal factors which influence a person’s natural predisposition when interacting with others [1]. Internal factors include brain development and integrity, personality and temperament and are often conceptualised within the domain of cognitive skills. External factors have been described to comprise environmental influences, such as: family factors [35], parent behaviours [36], socioeconomic status (SES) [37] and culture [38]. Finally there is agreement that the mediating effect of the stated internal and external factors facilitate the product of either well-developed social functioning or result in maladaptive social behaviours [1, 19]. Surprisingly, little is known about the contextual factors and contexts within which social interaction occur, which may either inhibit or facilitate social functioning.

#### The influence of contextual factors on social functioning

The contexts within which the social interaction occurs (e.g., school, home, work) may also influence the way in which the individual interacts with others, as well as the nature and quality of social interactions [39, 40]. Despite research having a strong focus on adaptive behaviour [16], there has been limited research into the influence of contextual factors on a person’s social functioning; perhaps one of the most neglected areas in contemporary conceptual models [1, 33].

### Assessments of Social Functioning

In recognition of the importance of social functioning, the assessment and treatment of social difficulties has become a focus of research over past decades; particularly in the area of developmental disabilities [19, 41, 42]. Given the array of skills subsumed within the domain of social functioning, some measures attempt to broadly address these different domains, while others specifically target the assessment of a sub-set of skills [19].

The assessments examining social functioning are mostly in the form of pen-and-paper questionnaires with the majority of identified assessments relying on child self-report, while some revert to using peers, parents and teachers in proxy reporting [19]. Reliance on self-report alone is problematic due to: poor construct and/or criterion validity (low correlations with other assessments), susceptibility to social desirability, and reliance on a child’s ability to comply with instructions [19, 43]. Further, one-time parent and teacher ratings have been described as inadequate methods of measurement, as they solely rely on the objectiveness of the rater and perform poorly in capturing behavioural changes over time, across contexts and between different interactants [19, 44].

In addition to the form of the assessment, it is important for the measure to be based on a sound theoretical model and for the underlying psychometric properties of the assessment to be evaluated [1]. Measures can have different prognostic and/or analytical functions. Measures can be prognostically used to: a) predict a later outcome; b) determine suitability for a particular intervention; c) report on the responsiveness to a particular intervention; or d) determine the amount of intervention required (dosage) [45]. Measures may also be used analytically to: a) explain or understand the contexts; b) classify or identify subgroups of patients; c) allow exploration of relationship between factors; d) detect within subject change or between subgroup differences; and e) enable comparison of patients to other population subgroups or norms [45]. If an outcome measure is used to evaluate changes in clients over time following a particular intervention, the quality of responsiveness becomes important. Conversely if the measure is used as a screening measure to accurately diagnose the presence or absence of a condition, identification accuracy and therefore the interpretability of the measure is of primary concern as it indicates the overall precision of making a diagnosis [46].

### Reviews of Social Functioning Measures

In response to the plethora of assessments measuring social functioning, four reviews aiming to provide an overview of these measures have been conducted [9, 19, 47, 48]. While these reviews provided valuable information on some of the many assessments focusing on social functioning, they have several limitations. Two of the earlier reviews by Demaray et al. [47] and Merrell [48] only evaluated a small number of measures and the review by Matson and Wilkins [9] did not adopt a systematic design [19]. However, a more recent systematic review by Crowe et al. [19] built on the review by Matson and Wilkins [9]. Crowe et al. [19] evaluated 86 assessments under the broad domain of social functioning. The review focused on: fairly recently published assessments (i.e., 1988–2010), whether psychometric properties were reported and the popularity of the measures (i.e., number of citations) [19]. Therein lies the main limitation of the Crowe et al. [19] review in that they only reported on whether and if authors reported their measure’s psychometric properties, but the review did not report on all psychometric properties. Moreover, the review lacked rigor in evaluating the quality of the psychometric properties of the measures in a systematic and uniform manner.

### Limitations of Current Assessments Measuring Social Functioning

Across the literature, several limitations to current social functioning assessments have been identified. These limitations include discrepancies regarding the definition of social functioning and subsequently a lack of connection between measures to a theoretical model [19, 49]. We add two further limitations to current social functioning assessments: 1) a lack of observational assessments, and 2) lack of uniform reporting on the psychometric properties of these assessments.

#### Definitions of social functioning

Many assessments lack a clear connection to a theoretical framework and clear definitions of the domains of social functioning being measured. Under the umbrella term *social functioning* are the following constructs which are often used interchangeably: pro-social behaviour [50], social adjustment [12], social cognition [51], social competence [52], social outcomes [53] and social skills [54]. The discrepancies present several challenges to scientific literature; including comparisons across studies, evaluation of the quality of assessments being used and most importantly, barriers to determining the effectiveness of treatments aiming to ameliorate difficulties surrounding social functioning [1, 49].

#### Lack of observational assessments

There is a near complete absence of well validated measures that assess social functioning through direct observation. Observational measures may provide numerous benefits including a social and ecologically valid approach, whereby individuals can be assessed performing the skills within the contexts they experience the difficulties. However, the few observational assessments that currently exist are study-specific, unpublished or were modified from adult or aggression measures [19].

#### Uniform reporting of psychometric properties

Evident within this area of research are lack of uniform reporting on both the description and psychometric properties of such measures. As noted by Crowe and colleagues [19], the literature on the psychometric properties of assessments of social functioning is continually being updated. Further reviews are needed to update and provide a comprehensive review into the psychometric properties of the available assessments.

### Study Aim

The purpose of this systematic review was twofold. Firstly, this review aimed to provide an overview of information on existing assessments that measure areas of social functioning across the lifespan; highlighting current gaps in the age, type, or context in which assessments can be administered. Within the area of social functioning, we focused the review to assessments of social skills and social behaviour.

Secondly, within this review, a central aim was to comprehensively evaluate the quality of psychometric properties reported across these assessments. To guide this aim, we used the COSMIN taxonomy of measurement properties and definitions for health-related patient-reported outcomes [55].

## Methods

The PRISMA statement was used to guide the methodology and reporting of this systematic review. The PRISMA statement checklist contains a total of 27 item areas that are deemed essential for the transparent reporting of systematic reviews [56]. A completed PRISMA checklist applicable to the current review is accessible (see Table in S1 Table).

### Eligibility Criteria

Eligibility criteria for studies in this review included research articles or published manuals on the psychometric properties of instruments designed to measure the social skills and behaviours of the general population. We adopted the following, widely used definition of social skills to guide our review, which comprises both skills and behavioural elements that result in positive social interactions, encompassing: 1) cooperation, 2) verbal and non-verbal communication, 3) engagement and participation, 4) empathy, and 5) self-regulation and adaptive behaviours in situations where interpersonal interaction occurs [16, 57]. Within this search, instruments measuring these skills and behaviours in both children and adults were included. For instruments to be included in this review, their main components or subscales needed to meet the definition we adopted of social skills. Instruments or published articles written in languages other than English were not eligible. As we were interested in evaluating the quality of psychometric properties of contemporary measures being used in recent research, instruments were excluded if they were published before 1994. For the purpose of this review, instruments that had an update of their psychometric properties in the last 20 years at the time of the search were regarded as contemporary. Instruments were further excluded if they were developed for a specific target population (e.g., autism spectrum disorders) rather than a normative sample. Articles were excluded if no psychometric properties were reported. Conference abstracts, reviews, case reports, student dissertations and editorials were also excluded.

### Information Sources

A systematic literature review was performed using four electronic databases: CINAHL, PsycINFO, Embase and Medline. Furthermore, Ovid's Health and Psychosocial Instruments (HAPI) database was used to identify potential instruments that met the inclusion criteria. The HAPI database provides access to information on instruments relevant to health related disciplines, including the fields of social sciences, organisational behaviour, and library and information sciences. Database searches were conducted between 3/05/2014 and the 15/05/2014. Search strategies included both free text words and subject headings (see Table 1), and comprised all journal articles up to May 2014. The second author conducted the searches because of her expertise in conducting systematic reviews. The databases were accessed from the libraries of Curtin University and James Cook University.

**Table 1:** Search Terms.

	Database and Search Terms	Limitations
**Subject Headings**	**CINAHL**: ((MH “Psychometrics”) OR (MH “Measurement Issues and Assessments”) OR (MH “Reliability”) OR (MH”Interrater Reliability”) OR (MH “Reliability and Validity”) OR (MH Test-Restest Reliability”) OR (MH “Intrarater Reliability”) OR (MH “Criterion-Related Validity”) OR (MH “Validity”) OR (MH “Predictive Validity”) OR (MH “Internal Validity”) OR (MH “Face Validity”) OR (MH “External Validity”) OR (MH “Discriminant Validity”) OR (MH “Consensual Validity”) OR (MH “Concurrent Validity”) OR (MH “Qualitative Validity”) OR (MH “Construct Validity”) OR (MH “Content Validity”) OR (MH “Measurement Error”) OR (MH”Bias (Research)”)) AND ((MH “Social Skills”) OR (MH “Social Skills Training”) OR (MH “Social Interaction Skills (IowaNOC)”) OR (MH “Social Behavior Disorder”) OR (MH “Social Behavior”) OR (MH “Social Adjustment”) OR (MH “Social Attitudes”) OR (MH “Social Participation”) OR (MH “Social Problems”)) AND ((MH “Outcome Assessment”) OR (MH “Behavior Rating Scales”) OR (MH “Social Readjustment Rating Scale”) OR (MH “Psychological Tests”) OR (MH “Health Screening”) OR (MH “Patient Assessment”) OR (MH “Self Assessment”) OR (MH “Measurement Issues and Assessments”) OR (MH “Scales/ED/MT/ED”) OR (MH “Questionnaires/EV/MT/ED) OR (MH “Clinical Assessment Tools”))	English Language; Human
	**PsycINFO**: (Psychometrics/ OR statistical reliability/ OR statistical validity/ OR “error of measurement”/) AND (social skills/ OR social reinforcement/ or social responsibility/ OR social acceptance/ OR social adjustment/ OR social anxiety/ OR social approval/ OR social behavior/ OR social cognition/ OR social comparison/ OR social control/ OR social deprivation/ OR social desirability/ OR social discrimination/ OR social skills training/ or social stress/ or social structure/ or social support/ or social values/) AND (Measurement/ OR questionnaires/ or rating/ OR rating scales/ OR psychological screening inventory/ OR screening/ OR surveys/)	English language; Human
	**Embase:** (Validation study/ OR validity/ OR psychometry/ OR reliability/ OR measurement accuracy/ OR measurement error/ OR measurement precision/ OR measurement repeatability/) AND (social acceptance/ OR social adaptation/ OR social bonding/ OR social change/ OR social cognition/ OR social cognitive theory/ OR social competence/ OR social adaptation/ OR social learning/ OR social learning theory/ OR social life/ OR socialization/ OR social behavior/ OR social stress/ OR social support/ OR social desirability/ OR social attitude/ OR social disability/ OR social exclusion/ OR social interaction/ OR social participation/ OR social phobia/ OR social problem/ OR social rejection/ OR social validity/) AND (questionnaire/ OR rating scale/ OR psychological rating scale/ OR screening/ OR screening test/ OR outcome assessment/ OR “social and occupation functioning assessment scale”/ OR social interaction anxiety scale/ OR social interaction test/ OR social support index/ OR “named inventories, questionnaires and rating scales”/ OR social phobia scale/ OR social readjustment rating scale/ OR social recognition test/ OR social support index/)	English language; Human
	**Medline:** (Psychometrics/) AND (Questionnaires/ OR Treatment Outcome/ OR “Outcome Assessment (Health Care)”/ OR Neuropsychological Tests/ OR Psychological Tests/) AND (Social behavior/ OR social participation/ or Social Problems/ OR Social Behavior Disorders/ OR social adjustment/ OR Emotions/)	English language; Human
**Free Text Words**	**CINAHL:** (social* OR emotion*) AND (psychometric* OR reliability OR validit* OR reproducibility OR bias) AND (questionnaire* OR assessment* OR test OR tests OR evaluation*)	Publication Date: 20130401–20130631 English Language; Human
	**PsycINFO:** *As per CINAHL Free Text*	(No Related Terms) yr = "2014-Current" and English language and human
	**Embase:** *As per CINAHL Free Text*	(No Related Terms) yr = "2014-Current" and english language and human
	**Medline:** *As per CINAHL Free Text*	yr = "2013-Current" and English language and human

We searched for grey literature using Google Scholar and PsycEXTRA. PsycEXTRA is the American Psychological Association's grey literature database which accompanies the PsycINFO database. It combines bibliographic records with full-text professional and lay-audience literature in the behavioural and social sciences. To be comprehensive, we also searched the websites of three major publishers of assessments in social sciences (Pearson, Acer and Western Psychological Services) to identify potential assessment not identified in earlier search strategies.

### Literature Search

A total of 2,117 abstracts were retrieved, including duplicates. The total abstracts from free text words and subject headings searches across each database were: CINAHL = 574, PsycINFO = 186. Embase = 711, Medline = 646. A total of 220 duplicates across the four databases were removed. The electronic search strategy used for each database, including: subject heading, free text and limitations are reported in Table 1. Reference lists of the included articles were searched for additional literature.

**Fig 1:**
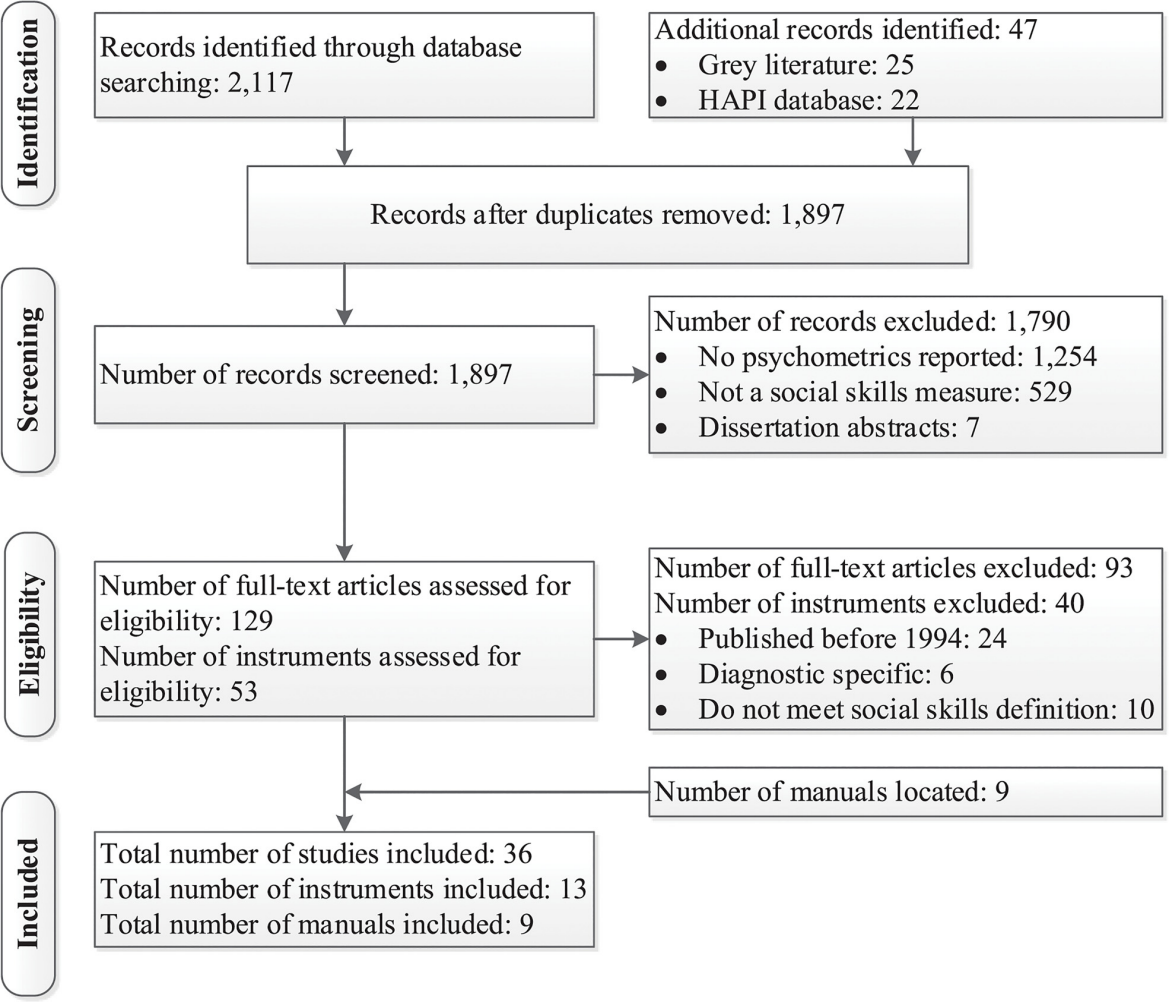
Flow diagram of the reviewing process according to PRISMA. Adapted from Moher et al. [58].

The HAPI database identified 22 instruments that potentially met the inclusion criteria; thus warranting further scrutiny. Search of grey literature identified an additional 25 records; thus a total of 47 records were identified. Fig 1 presents the flow diagram of the reviewing process according to PRISMA [58].

### Study Selection

Two independent abstract reviewers rated the abstracts on the following inclusion criteria: abstracts had to describe an instrument or outcome measure; address its psychometric measurement properties; and assess social skills and behaviours. A random sample of 40% of the abstracts was examined to determine the inter-rater reliability: Weighted Kappa = 0.79 (95% CI 0.72–0.86). To ensure all instruments measured social skills and/or social behaviour, two doctoral candidates with knowledge in the area of social skills reviewed the instruments together. At this level, the abstracts of the articles and descriptions of the assessment were located to ensure the instrument met the adopted definition of social skills [16, 57].

### Data Collection Process / Data Extraction

To capture the data contained within the included studies and manuals (45) we used the Cochrane Handbook for Systematic Reviews section 7.3a [59], and the Systematic Reviews Centre for Reviews and Dissemination [60]. Data were extracted under the following headings: study design, purpose of the study, study population, age of the population, and instrument characteristics. To both capture the data and assess the methodological quality of the data the COSMIN was used [55].

### Methodological Quality

The psychometric quality of the included instruments were then analysed using the COSMIN taxonomy of measurement properties and definitions for health-related patient-reported outcomes [61]. The COSMIN checklist [55] is a standardised tool for assessing the methodological quality of studies on measurement properties and consists of nine domains: internal consistency, reliability (relative measures: including test-retest reliability, inter-rater reliability and intra-rater reliability), measurement error (absolute measures), content validity (including face validity), structural validity, hypotheses testing, cross-cultural validity, and criterion validity. Responsiveness as a psychometric property was not evaluated in this review. Definitions of all the psychometric properties, as defined in the COSMIN statement, are provided in Table 2. Interpretability is not considered to be a psychometric property under the COSMIN framework and was therefore not described in this review. Each domain of the COSMIN checklist includes 5 to 18 items focussing on different aspects of study design and statistical analyses. Terwee et al. [62] proposed using a 4-point rating scale per item (excellent, good, fair, and poor), obtaining an overall methodological quality score per psychometric property by taking the lowest rating of any item in the corresponding domain. As this rating system appears to be so severe that it inhibits differentiation between more subtle psychometric qualities of instruments [63], a revised scoring was introduced. The outcome was presented as percentage of rating (Poor = 0–25.0%, Fair = 25.1%-50.0%, Good = 50.1%-75.0%, Excellent = 75.1%-100.0%). Given that some COSMIN items only have excellent and good as an option for rating, we calculated the total score for each psychometric property using the following formula to most accurately capture the quality of the psychometric properties:

$$\text{Total score for psychometric property}=\frac{\left(\text{Total score obtained}-\text{minimum score possible}\right)}{\left(\text{Max score possible}-\text{minimum score possible}\right)}\;\text{X}\;100$$

To ensure consistency of the COSMIN checklist ratings, the first author trained two independent doctoral candidates to complete the COSMIN checklist. To ensure accuracy, the two raters completed COSMIN checklists together for 10 of the 13 instruments.

**Table 2:** COSMIN: Definitions of Domains, Psychometric Properties, and Aspects of Psychometric Properties for Health-Related Patient-Reported Outcomes based on Mokkink et al. [61]

Psychometric property	Domain and Definition^a^
	**Reliability:** the degree to which the measurement is free from measurement error.
Internal consistency	The degree of the interrelatedness among the items.
Reliability	The proportion of the total variance in the measurements which is because of “true” differences among patients.
Measurement error	The systematic and random error of a patient’s score that is not attributed to true changes in the construct to be measured.
	**Validity:** the degree to which an instrument measures the construct(s) it purports to measure.
Content validity	The degree to which the content of an instrument is an adequate reflection of the construct to be measured.
**Face validity** ^**b**^	The degree to which (the items of) an instrument indeed looks as though they are an adequate reflection of the construct to be measured.
Construct validity	The degree to which the scores of an instrument are consistent with hypotheses based on the assumption that the instrument validly measures the construct to be measured.
**Structural validity** ^**c**^	The degree to which the scores of an instrument are an adequate reflection of the dimensionality of the construct to be measured.
**Hypothesis testing** ^**c**^	Item construct validity.
**Cross-cultural validity** ^**c**^	The degree to which the performance of the items on a translated or culturally adapted instrument are an adequate reflection of the performance of the items of the original version of the instrument.
Criterion validity	The degree to which the scores of an instrument are an adequate reflection of a “gold standard”.
Responsiveness	**Responsiveness:** the ability of an HR-PRO instrument to detect change over time in the construct to be measured.
Interpretability^d^	**Interpretability** ^a^: the degree to which one can assign qualitative meaning to an instrument’s quantitative scores/ score change.

Notes.

^a^Applies to Health-Related Patient-Reported Outcomes (HR-PRO) instruments.

^b^Aspect of content validity under the domain of validity.

^c^Aspects of construct validity under the domain of validity.

^d^Interpretability is not considered a psychometric property

### Data Items, Risk of Bias and Synthesis of Results

All data items for each instrument were obtained. When an item was not reported, an ‘NR’ was recorded. Risk of bias was assessed at an individual study level during the rating of the COSMIN checklist through the inclusion of ‘methodological limitations items’. The results were synthesised and grouped as follows: 1) development and validation of the instrument, 2) the psychometric properties of the instruments, and 3) the instrument characteristics.

## Results

### Systematic Literature Search

After the removal of duplicate abstracts across the four databases, a total of 1,897 studies were screened for inclusion in this review. Of these studies, 129 full-text articles on 53 measures were assessed for eligibility (see Fig 1). Of these 53 measures, 13 measures met the inclusion criteria and 40 were excluded for the following reasons: 24 were published before 1994, 6 were diagnostic specific, and 10 did not meet the definition of social skills adopted for the purpose of this review. See Table 3 for an overview of the 40 social skills instruments and the reasons for exclusion. Through additional searches another 9 manuals were located. Thus, the psychometric properties were obtained for a total of 13 social skills measures which were accessed using 36 articles and 9 manuals.

**Table 3:** Overview of Social Skills Instrument: Reasons for Exclusion.

	Instrument	Acronym	Reason for Exclusion
1.	Aberrant Behavior Checklist [64]	ABC-ID	Published before 1994; target population: intellectual disabilities;
2.	Alarme Distress de Bebe Scale (Alarm Distress Baby scale) [65, 66]	ADBB	Did not meet social skills definition
3.	Anticipatory Social Behaviours Questionnaire [67]	ASBQ	Did not meet social skills definition
4.	Autism Behavior Checklist [68]	ABC	Target population: ASD
5.	Autism Social Skills Profile [69]	ASSP	Target population: ASD
6.	Behavioral Assessment System for Children (BASC) [70]	BASC	Published before 1994
7.	Behavioral Repertoire Self-Rating Questionnaire [71]	BRSRQ	Published before 1994; did not meet social skills definition
8.	Blushing, Trembling, and Sweating Questionnaire [72]	BTSQ	Did not meet social skills definition; Target population: Phobias
9.	Child Behavior Rating Scale [73–75]	CBRS	Published before 1994
10.	Children’s Action Tendency Scale [76, 77]	CATS	Published before 1994
11.	Children’s Assertive Behavior Scale [76, 78]	CABS	Published before 1994
12.	Children's Social Behavior Questionnaire [79–82]	CSBQ	Target population: ASD
13.	Community-based Social Behavior Assessment Battery [83]	CBSB	Target population: emotional and behavioral disorders
14.	Conflict Behavior Questionnaire [84]	CBQ	Published before 1994; did not meet social skills definition
15.	Coping Inventory for Stressful Situations [85]	CISS	Published before 1994; did not meet social skills definition
16.	Experienced Social Mobility Questionnaire [86]	ESMQ	Did not meet social skills definition
17.	General Health Questionnaire-12 [87]	GHQ-J	Published before 1994; did not meet social skills definition;
18.	Maternal Social Support Index [88]	MSSI	Published before 1994; did not meet social skills definition
19.	Minimal social behavior scale [89, 90]	MSBS	Did not meet social skills definition; published before 1994
20.	Personality Disorder Questionnaire—Revised [91]	PDQ-R	Published before 1994
21.	Personal and Social Performance Scale [92]	PSP	Did not meet social skills definition; target population: psychiatric population
22.	Scale of Negative Social Exchanges [93]	SNSE	Did not meet social skills definition
23.	Self-Reported Antisocial Behavior Questionnaire [94]	SRABQ	Published before 1994; target population: ADHD
24.	School Social Skills Rating Scale [47, 95]	S-3	Published before 1994
25.	Social Adjustment Rating Scale—Self-Report [96]	SAS-SR	Published before 1994
26.	Social Behavior Inventory [97]	SBI	Target population: child abuse
27.	Social Behavior Assessment Inventory [47, 98]	SBAI	Published before 1994
28.	Socially Desirable Response Set [99]	SDRS	Published before 1994; did not meet social skills definition
29.	Social Influences Scale [100]	SIS	Published before 1994; did not meet social skills definition
30.	Social Phobia and Anxiety Inventory [101]	SPAI	Published before 1994; did not meet social skills definition
31.	Social Problems Questionnaire [102]	SPQ	Published before 1994
32.	Social Readjustment Rating Scale-modified [103]	SRRS-M	Published before 1994; target population: emotional and behavioral disorders
33.	Social Role Competence Measure [104]	SRCM	Did not meet social skills definition; No psychometrics
34.	Social Skills Inventory [105, 106]	SSI	Published before 1994
35.	Subtypes of Antisocial Behavior Questionnaire [107]	STAB	Did not meet social skills definition
36.	Teenage Inventory of Social Skills [108, 109]	TISS	Published before 1994
37.	The Social Skills Q-Sort [110]	SSQ	Target population: ASD
38.	Waksman Social Skills Rating Scale [47, 111]	WSSRS	Published before 1994
39.	Walker-McConnell Scale of Social Competence and School Adjustment [47, 112]	WMS	Published before 1994
40.	Young Adult Social Behavior Scale [113]	YASB	Did not meet social skills definition

*Note*. ASD = Autism Spectrum Disorders; ADHD = Attention Deficit Hyperactivity Disorder.

### Included Social Skills Measures

Information on the development and validation of the 13 included social skills measures is reported in Table 4. All measures had some evidence of development and validation through the use of a normative study population/sample; without targeting a specific diagnostic group. Of the 13 measures, 12 were developed using children up to 12 years of age; with 6 of these measures also using an adolescent sample (13–18 years). No measure was developed using an adult population alone (older than 18 years) and only 1 measure (i.e., the Evaluation of Social Interaction [ESI]) was developed and validated using all three age groups (i.e., children, adolescents and adults)

**Table 4:** Description of Studies for the Development and Validation of Instrument for the Assessment of Social Skills.

Instrument	Reference	Purpose of study	Study population	Age (range[R] and/or Mean [M] Standard deviation [SD])
*Evaluation of Social Interaction (ESI)*	Simmons et al. [114]	Internal scale validity, items' skill hierarchy and intended purpose, differentiation between people with and without disability	***N* = 134** *(I)* 60–73y age group; *(II)* 35–59y age group; *(III)* 17–34y age group; *(IV)* 4–15y age group. Number of participants in each group is not reported; population characteristics are not pecified.	*Total sample*: R = 4–73y; M = NR; SD = NR. *(I)* R = 60–73y; M = 65.4y; SD = NR. *(II)* R = 35–59y; M = 45.2y; SD = NR. *(III)* R = 17–34y; M = 24.9y; SD = NR. *(IV)* R = 4–15y; M = 6.5y; SD = NR.
	Søndergaard and Fisher [115]	Sensitivity to differentiate between people without identified diagnosis and those with neurologic or psychiatric disorders	***N* = 185** (I) control: *n* = 304; *(II)* neurologic: *n =* 77; *(III)* psychiatric: *n =* 104	*Total sample*: R = 16y-69y; M = NR; SD = NR. *(I)* R = NR; M = 41.9y; SD = 13.6y. *(II)* R = NR; M = 44.9y; SD = 11.9y. *(III)* R = NR; M = 35.5 SD = 11.1y.
	Griswold and Townsend [116]	Sensitivity to differentiate between children with and without disabilities	***N* = 46** *(I)* with disabilities: *n =* 23; *(II)* matched without disabilities: *n =* 23	*Total sample*: R = 2y-12y; M = 7y; SD = NR. *(I)* R = NR; M = NR; SD = NR. *(II)* R = NR; M = NR; SD = NR.
	Fisher and Griswold [117]	Development and validation	***N* = 6,552** (I) well: *n =* 4,099; *(II)* at risk/mild: *n =* 235; *(III)* psychiatric: *n =* 915; *(IV)* developmental: *n =* 320; *(V)* neurological: *n =* 300; *(VI)* other/ multiple: *n =* 682	*Total sample*: R = 2y-80+y; M = NR; SD = NR. *(I)* R = NR; M = NR; SD = NR. *(II)* R = NR; M = NR; SD = NR. *(III)* R = NR; M = NR; SD = NR. *(IV)* R = NR; M = NR; SD = NR. *(V)* R = NR; M = NR; SD = NR. *(VI)* R = NR; M = NR; SD = NR.
*Home and Community Social Behavior Scales (HCSBS)*	Merrell et al. [118]	Convergent and discriminative validity. *Study 1*: Comparisons with the Social Skills Rating System and Conners Parent Rating Scales; *Study 2*: Comparisons With the Child Behavior Checklist; and *Study 3*: Comparisons With the Behavior Assessment System for Children.	***N* = 325** *Study 1*: *n =* 59; *Study 2*: *n =* 60; *Study 3*: *=* 206 *(I) n =* 76 *(II) n =* 130	*Total sample*: R = NR; M = NR; SD = NR. *Study 1*: Grade 6 (R = NR; M = NR; SD = NR). *Study 2*: Grade 2–5 (R = NR; M = NR; SD = NR). *Study 3*: *(I)* R = 6–12y; M = NR; SD = NR *(II)* R = 12–18y; M = NR; SD = NR.
	Lund and Merrell [119]	Construct validity to discriminate sensitivity of group differences	***N* = 180** *(I)* emotional-behavioral disorders: *n =* 60 *(II)* learning disabilities: *n =* 60 *(III)* without disabilities: *n =* 60	*Total sample*: R = 6y-12y; M = NR; SD = NR. *(I)* R = NR; M = NR; SD = NR. *(II)* R = NR; M = NR; SD = NR. *(IIII)* R = NR; M = NR; SD = NR.
	Merrell and Caldarella [120]	Internal consistency, correlations among subscales, discrimination between groups (at-risk vs. non-risk)	***N* = 267 *(I)* non-risk: *n =* 107;** *(II)* at-risk: *n =* 160	*Total sample*: R = 11y-16y; M = NR; SD = NR. *(I)* R = NR; M = NR; SD = NR. *(II)* R = NR; M = NR; SD = NR.
	Merrell and Caldarella [120]	Development and validation of HCSBS	***N* = 1,562** School aged children	*Total sample*: R = 5y-18y; M = NR; SD = NR.
*Interaction Rating Scale (IRS)*	Anme et al. [121]	Describe the features of IRS as an evidence-based practical index of children's social skills and parenting difference in age skills)	***N* = 814** *(I) n =* 213 (18m age group); *(II) n =* 344 (30m age group); *(III) n =* 175 (42m age group); *(IV) n =* 82 (7y age group)	*Total sample*: R = 18m-7y; M = NR; SD = NR. *(I)* R = NR; M = 18m; SD = NR. *(II)* R = NR; M = 30m; SD = NR. *(III)* R = NR; M = 42m; SD = NR. *(IV)* R = NR; M = 7y; SD = NR.
*Interaction Rating Scale Advanced (IRS–Advanced)*	Anme et al. [122]	Validity of IRS-A as an evidence-based practical index of social competence	***N* = 17** High school students	*Total sample*: R = 16–17y; M = NR; SD = NR.
	Anme et al. [123]	Validity and reliability of the IRS-A	***N* = 50** Adults	*Total sample*: R = 18–48y; M = NR; SD = NR.
*Interaction Rating Scale (IRS–BC)*	Anme et al. [124]	Validate the IRS-BC.	***N* = 20** Children	*Total sample*: R = 5–6y; M = NR; SD = NR.
*Matson Evaluation of Social Skills with Youngsters-II (MESSY–II)*	Matson et al. [125]	Internal consistency in different age group, convergent and divergent validity. *Study 1*: Assess the reliability and validity of the MESSY parent-report form using an updated sample of typically developing children *Study 2*: Examine internal consistency reliability, convergent and divergent validity.	***N* = 1,138** *Study 1*: *(I) n =* 286 (2–5y age group); *(II) n =* 301 (6–9y age group); *(III) n =* 298 (10–16y age group); Study 2: *(I) n =* 78 (2–5y age group) *(II) n =* 130 (6-9y age group) *(III) n =* 45 (10–17y age group)	*Total sample*: R = NR; M = NR; SD = NR. *Study 1*: *(I)* R = 2–5y; M = 3.9y; SD = 1.0y. *(II)* R = 6-9y; M = 7.4y; SD = 1.1y. *(III)* R = 10–16y; M = 12.3y; SD = 1.8y. *Study 2*: *(I)* R = 2–5y; M = 4.2y; SD = 0.9y. *(II)* R = 6–9y; M = 7.20y; SD = 1.2y. *(III)* R = 10–17y; M = 10.9y; SD = 1.0y.
	Matson et al. [126]	Factor structure and internal consistency	***N* = 886** Typically developing children	*Total sample*: R = 2–16y; M = 7.9y; SD = 3.7y.
	Méndez et al. [127]	Psychometric properties of the Spanish translation of MESSY	***N* = 634** Adolescents	*Total sample*: R = 12–17y; M = 14.3; SD = 1.7.
	Teodoro et al. [128]	Psychometric properties of the Brazilian version of MESSY	***N* = 382** Children	*Total sample*: R = 7–15y; M = 10.3y; SD = 1.6y.
	Chou [129]	Psychometric properties of the Chinese translation of MESSY	***N* = 191** Children	*Total sample*: R = 11–18y; M = 14.0; SD = 1.6y
	Matson et al. [130]	*(I)* internal consistency, split half reliability *(II)* inter-rater reliability	***N* = 147** *(I)* children with ASD/ PDD-NOS: *n =* 114; *(II)* children with ASD/ PDD-NOS: *n =* 33 (participants a subset of the children from study I)	*(I)* R = 2–16y; M = 7.5y; SD = 3.3y. *(II)* R = 5–16y; M = 9.1y; SD = 3.1y.
*Preschool and Kindergarten Behavior Scales 2 (PKBS-2)*	Merrell [131]	Development and validation of the instrument PKBS-2	***N* = 3,258** Community sample: *n =* 286 (3y age group); *n =* 577 (4y age group); *n =* 1,194 (5y age group; *n =* 1,201 (6y age group)	*Total sample*: R = 3–6y; M = NR; SD = NR.
	Merrell [132]	Relationship between social skills and internalizing problems (PKBS)	***N* = 2,455** Internalizing at-risk group: *n =* 193 children; Non-internalizing group: *n =* 2262	*Total sample*: R = NR; M = NR; SD = NR.
	Merrell [133]	Convergent and discriminant construct validity of PKBS	***N =* 295** *Study 1*: children who had been referred to a specific education child find program: *n =* 86; *Study 2*: developmentally delayed: *n =* 116; *Study 3*: kindergarten students: *n =* 46; *Study 4*: kindergarten students: *n =* 47	*Total sample*: R = NR; M = NR; SD = NR. *Study 1*: R = 3–6y; M = NR; SD = NR. *Study 2*: R = 3–6y; M = NR; SD = NR. *Study 3*: R = 5–6y; M = NR; SD = NR. *Study 4*: R = 5–6y; M = NR; SD = NR.
	Canivez and Rains [134]	Construct validity	***N =* 124** *(I)* normal: *n =* 107; *(II)* disabled/at-risk: *n =* 17	*Total sample*: R = 5–6y; M = 6.2y; SD = 0.4y (*I*) R = NR; M = NR; SD = NR (*II*) R = NR; M = NR; SD = NR
	Canivez and Bordenkircher [135]	Convergent and divergent validity of the PKBS	***N =* 154** *(I)* typical: *n =* 137; *(II)* disabled/at-risk: *n =* 17	*Total sample*: R = 5–6y; M = 5.4y; SD = 0.5y. *(I)* R = NR; M = NR; SD = NR. *(II)* R = NR; M = NR; SD = NR.
	Carney and Merrell [136]	Reliability and comparability of a Spanish-language form of the PKBS	***N =* 45** Children fluent in both English and Spanish	*Total sample*: R = 3–6y; M = NR; SD = NR.
	Wang et al. [137]	Psychometric properties used in ASD population; detecting change in social behavior of children with ASD in an early education program (PKBS)	***N =* 22** Children with ASD	*Total sample*: R = NR; M = NR; SD = NR. At pre-test: R = 36–76m; M = 56.5m; SD = NR. *At post-test*: R = 42–83m; M = 63.3m; SD = NR.
	Merrell [138]	Development of the PKBS	***N =* 2,885** Standardized sample, including: *(I)* previously identified as being developmentally delayed: *n* = 239; *(II)* currently being identified with disabilities: *n* = 73	*Total sample*: R = 3–6y; M = 56.5m; SD = NR.
	Carney and Merrell [139]	Construct validity in differentiating ADHD and non-ADHD (PKBS)	***N* = 60** *(I)* ADHD: *n =* 30; *(II)* comparison group: *n =* 30	*Total sample*: R = 60–83m; M = 56.5m; SD = NR. *(I)* R = NR; M = 74.8m; SD = 6.6m. *(II)* R = NR; M = 73.2m. SD = 6.0m.
	Fernández et al. [140]	Structural validity of Spanish language version of PKBS-2	***N* = 1,509** Preschoolers	*Total sample*: R = 3–5y; M = 3.8y; SD = 0.8y.
	Al Awmleh and Woll [141]	Reliability of German language versions of PKBS	***N* = 37** Preschoolers	*Total sample*: R = NR; M = 4.5y; SD = 0.7y.
	Edwards et al. [142]	Unidimensionality and reliability of PKBS	***N* = 1,679** Children from a program designed to integrate behavioral health services for children at risk (due to low income) into early childhood education or pediatric service settings	*Total sample*: R = 25–73m; M = 47.7m; SD = 7.2m.
*Peer Social Maturity Scale (PSMS)*	Fink et al. [143]	Convergent validity, internal consistency, reliability, factor analysis. *Study 1*: convergent validity. *Study 2*: reliability, confirmatory factor analysis, convergent validity.	***N* = 454** Children without developmental disabilities. *Study 1*: *(I)* Kindergarten: *n =* 54 *(II)* Grade 1: *n =* 44 *(III)* Grade 2: *n =* 40; *Study 2*: *Phase 1*: *n =* 114 *Phase 2*: *n =* 106 *Phase 3*: *n =* 96	*Total sample*: R = NR; M = NR; SD = NR. *Study 1*: *Total sample*: R = NR; M = 6y6m; SD = 10.4m, *(I)* R = NR; M = 5y7m; SD = 4.3m, *(II)* R = NR; M = 6y7m; SD = 4.5m, *(III)* R = NR; M = 7y6m; SD = 4.9m. *Study 2*: *Phase 1*: R = 55–77m; M = 5y7m; SD = 5.1m, *Phase 2*: R = 70–90m; M = 6y8m; SD = 4.8m, *Phase 3*: R = 80–102m; M = 7y8m; SD = 4.6m.
*Questionario de Respostas Socialmente Habilidosas Segundo Relato de Professores (QRSH-PR)*	Bolsoni-Silva et al. [144]	Content validity, internal consistency, construct validity, discrimination validity, concurrent validity, predictive validity	***N* = 260** *(I)* behavioral problems: *n =* 130; *(II)* social skilled behaviors: *n =* 130	*Total sample*: R = NR; M = NR; SD = NR. *(I)* R = NR; M = NR; SD = NR. *(II)* R = NR; M = NR; SD = NR.
*Social Competence Inventory (SCI)*	Rydell et al. [145]	Internal consistency, stability across one year, inter-rater agreement, discriminate peer status, factor analysis	***N* = 785** *(I)* pilot: *n =* 121; *(II)* Uppsala sample: *n =* 123; *(III)* provincial sample: *n =* 118; (IV) country sample: *n =* 423	*Total sample*: R = 7–10y; M = NR; SD = NR. *(I)* R = 7–10 y; M = NR; SD = NR. *(II)* R = NR; M = 7y11m; SD = 4m. *(III)* R = NR; M = 9y6m; SD = 4m. *(IV)* R = NR; M = 8y5m; SD = 3y3m.
*Social-Emotional Assets and Resilience Scales (SEARS)*	Merrell et al. [146]	Development and validation of SEARS (parent form)	***N* = 2,356** Children and adolescents, including 10.3% of the children with a disability and 89.7% without a disability	*Total sample*: R = 5–18y; M = NR; SD = NR.
	Merrell et al. [147]	Development and validation of SEARS (teacher form)	***N =* 1,673** Children and adolescents, including 17.7% of the students identified and eligible for special education services and 89.7% without a disability	*Total sample*: Kindergarten to Grade 12 (R = NR; M = NR; SD = NR).
	Romer and Merrell [148]	Temporal stability and test-retest reliability of SEARS (children, adolescent, and teacher rating)	***N =* 287** *(I)* student self-report sample: *n =* 169 students from general education social studies and science classrooms (49.1% for SEARS-C; 50.9% for SEARS-A); *(II)* teacher report sample: *n =* 118 students	*Total sample*: R = NR; M = NR; SD = NR. *(I)* Grade 6-Grade 8 (R = NR; M = NR; SD = NR). *(II)* kindergarten to Grade 5 (R = NR; M = NR; SD = NR).
	Merrell [149]	Validation of the instrument	***N =* 5,555** *(I)* SEARS-C normative sample: *n =* 1,224; *(II)* SEARS-A normative sample: *n =* 1,727; *(III)* SEARS-T normative sample: *n =* 1,400; *(IV)* SEARS-P normative sample: *n =* 1,204	*Total sample*: R = NR; M = NR; SD = NR. *(I)* R = 8–12y; M = 9.9y; SD = 1.2y. *(II)* R = 13–18y; M = 4.4y; SD = 1.9y. *(III)* children: R = 5–12y; M = NR; SD = NR; adolescents: R = 13–18y; M = NR; SD = NR. *(IV)* children: 5–12y; M = 8.7y; SD = 2.2y; adolescents: 13–18y; M = 15.3y; SD = 1.6y.
*Vineland Social-Emotional Early Childhood Scales (SEEC)*	Sparrow et al. [150]	Development and validation of the instrument	***N =* 1,200** Community sample across 36 US states; *n* = 200 in each 0–1y, 1y, 2y, 3y, 4y, & 5y age cohorts	*Total sample*: R = Birth-5y11m; M = NR; SD = NR.
*Social Profile (SP)*	Donohue [151]	Content validity, construct validity, inter-rater reliability	***N =* 187** Typically developing children	*Total sample*: R = 2–5y; M = NR; SD = NR.
	Donohue [152]	Inter-rater reliability	***N =* 35** *(I)* substance abuse group: *n =* 11; *(II)* general/geriatric group: *n =* 10; *(III)* preschool group: *n =* 12; *(IV)* senior group: *n =* 2	*Total sample*: R = NR; M = NR; SD = NR.
*School Social Behavior Scales-2 (SSBS-2)*	Merrell [17]	Development and validation of the instrument	***N =* 2,280** Students, including 10.7% with learning difficulty, 2.6% with speech or language impairment, 3.0% with intellectual disability, 4.1% with emotional disturbance, 1.9% with other disabilities, and 22.3% with all disabilities	*Total sample*: Grades K-12 (R = NR; M = NR; SD = NR).
	Raimundo et al. [153]	Analyze the psychometric properties of a Portuguese version of the social competence scale form the SSBS-2	***N =* 595** *Sample 1*: *n* = 344 students; *Sample 2*: *n* = 251 students	*Total sample*: R = NR; M = NR; SD = NR. *Sample 1*: R = 6–18y; M = 12.1y; SD = 3.4y. *Sample 2*: R = 8–14y; M = 9.3y; SD = 0.8y
*Social Skills Improvement System Rating Scales (SSIS)*	Gresham et al. [154]	Agreement of ratings among teachers, parents/caregivers, and students in SSIS	***N =* 332** Sample 1: *n =* 168 students; *(II) n =* 164 students	*Total sample*: R = NR; M = NR; SD = NR. *(I)* R = NR; M = 11.9y; SD = NR. *(II)* R = NR; M = 9y; SD = NR.
	Gresham and Elliott [155]	Development and validation	***N =* 4,700** Children and Adolescents aged 3–18y (Spanish sample: *n =* 486)	*Total sample*: R = 3–18y; M = NR; SR = NR

*Note*. BC = Between Children; m = months; NR = not reported; y = years.

The characteristics of the included measures are reported in Table 5. Of the 13 measures, 6 were published within the last 5 years (since 2009). Regarding the measure type, 9 measures used self-, parent- or teacher-report; with 3 of these measures using only teacher-report. Of the remaining measures, 3 were observation-based and only 1 used a semi-structured interview (see Table 5). Regarding the response options within the measures, 12 reported the use of Likert scales and only the ESI reported the use of a criterion-referenced rating scale. Of the 12 measures using a Likert response scale, 11 reported the use of a 3 to 5 point scale, and the Peer Social Maturity Scale (PSMS) reported the use of a 7-point scale. Additionally, the Interaction Rating Scale (IRS) reported the use of a dichotomous (yes or no) rating system for its scale.

**Table 5:** Characteristics of the Instrument for the Assessment of Social Skills.

Instrument	Purpose of instrument	Published year	Type of measure	Number of Subscales/ Forms	Number of items in total	Response Options
ESI [117]	To assess a person's performance of social interaction skills in the natural context with typical social partners during any area of occupation	2009/2014 (latest version)	Observation	1 scale	27	*4-point criterion-referenced rating scale*: 4 = socially appropriate, polite, respectful, and timely
HCSBS [156]	Assess social skills and antisocial behavior across environment	2000	Parent/ caregiver rating	2 subscales	65	*5 point scale*: 1 = never to 5 = frequently
IRS [121]	To measure child's social competence and the caregiver's child rearing competence in caregiver-child interactions	2009	Observation	5 subscales	70	*Behavior items*: 1 = yes or 0 = no. *5-point rating for impression items*: 1 = not evident at all; 2 = not evident; 3 = neutral; 4 = evident; 5 = evident at high level
IRS–Advanced [122]	An advanced version of IRS, measure social competence for individuals over 15 years old	2011	Observation	6 subscales	92	See IRS
IRS–BC [157]	A peer relationship version of the IRS, to measure child-child interaction	2012	Observation	3 subscales	43	See IRS
MESSY-II [125]	To measure both appropriate and inappropriate social behaviors for children	2010	Parent/ (teacher)/ (self-report) rating	1 scale; 3 forms	Parent/ (teacher) rating: 64	*5-point scale*: 1 = not at all to 5 = very much
PKBS-2 [131]	To measure social and emotional problems of children with significant behavioral, emotional and developmental problems	2003	Parent/teacher rating	2 scales	*Social Skill Scale*: 34 *Problem Behavior Scale*: 42	*4-point scale*: 0 = never; 1 = rarely; 2 = sometimes; 3 = often
PSMS [143]	To capitalize on teacher's experience with pupils of varying social competence	2007	Teacher rating	1 scale	7	*7-point rating*: from 1 = very much less mature than the average child this age to 7 = very much more mature than the average child this age
QRSH-PR [144]	To assess social skills in preschool children	2009	Teacher rating	1 scale	24	*3-point Likert scale*: 0 = definitely applies; 1 = applies a little; 2 = does not apply
SCI [98]	To measure social competence	1997	Parent/ teacher rating	2 subscales	29	*5-step response scale*: 1 = doesn't apply at all; 5 = applies very well to the child
SEARS [149]	To assess social and emotional characteristics of children and adolescents focuses on the affective, interpersonal, behavioral, and cognitive aspects of their adjustment	2011	Parent/ teacher/ children/ adolescent rating	4 forms	*Parent/ teacher rating*: 54 *Children/ adolescent rating*: 35	*4-point rating*: 0 = never to 3 = always
SEEC [150]	To assess usual social and emotional functioning for children from birth to 5 year 11 months	1998	Semi-structured interview	3 subscales	*Interpersonal relationship*: 44 *Play and leisure time*: 44 *Coping skills*: 34	0 = never performed; 1 = performed sometimes or with partial success; 2 = usually or habitually performed
SP [158]	To evaluate the social participation levels of children in activity groups	2005	Observation	3 sections/ 2 versions (children, adult / adolescent)	166	*5-point Likert scale* (for each level of social participation, 5 levels): 1 = never; 2 = rarely; 3 = sometimes; 4 = frequently; 5 = almost always
SSBS-2 [153]	To screen and assess social competence and antisocial behavior of students from K to 12 educational settings	2002	Teacher rating	2 Scales	*Social Competence* Scale: 32 *Antisocial Behavior Scale*: 33	1 = never to 5 = frequently
SSIS [155]	To measure social skills and problematic behaviors	2008	Self-rating / parent/ teacher rating	3 forms	*Teacher form*: 83 *Parent form*:79 *Student form*: 75	*4-point scale for skills*: never, seldom, often and almost always; *4-point scale for problems*: not true; a little true, a lot true; very true. *3-point scale for importance in skills*: important, not important, critical; *teacher form*: additional 5-point scale for academic competence

### Psychometric Properties

The quality ratings of the psychometric properties of all 13 measures, which were evaluated against the COSMIN quality criteria, are summarised in Table 6. The overall means and standard deviations of each psychometric property across all social skills measures were also calculated. S*tructural validity* was most frequently reported; the mean rating across 12 measures was 78.1 (SD = 14.8), indicating excellent quality. The least reported psychometric property was *criterion validity*; 3 measures had a mean rating of 80.7 (SD = 7.9), indicating excellent quality. Overall, 11 measures had evidence for *internal consistency*; the mean rating across these measures was 84.5 (SD = 12.9), indicating excellent quality. The mean rating of the 11 measures reporting on *reliability* and *hypothesis testing* was 74.7 (SD = 11.9) and 56.8 (SD = 17.5) respectively; indicating good quality. The mean rating of the 6 measures that reported on *measurement error* was 70.0 (SD = 12.8); indicating good quality. *Content validity* was reported by 8 measures; mean rating 74.0 (SD = 17.0), indicating good quality. Cross-cultural validity was reported by 4 measures; mean rating 54.3 (SD = 13.2), indicating good quality.

**Table 6:** Overview of the Psychometric Measurement Properties of Social Skills Instrument.

Instrument	Author(s)	Year	Internal consistency	Reliability	Measurement error	Content validity	Structural validity	Hypotheses testing	Cross-cultural validity	Criterion validity	Average
**ESI**	Simmons, Griswold [114]	2010^A^	Excellent (90.5)	-	-	-	Excellent (83.3)	Excellent (82.6)	-	-	NA
	Søndergaard and Fisher [115]	2012^A^	-	-	-	-	-	Excellent (82.6)	-	-	NA
	Griswold and Townsend [116]	2012^A^	-	-	-	-	-	Good (52.9)	-	-	NA
	Fisher and Griswold [117]	2014^M^	Excellent (100)	Good (62.1)	Good (61.4)	-	Excellent (100)	Excellent (90.9)	-	-	NA
	**TOTAL**	**NA**	**Excellent (95.2)**	**Good (62.1)**	**Good (61.4)**	**NR**	**Excellent (91.7)**	**Excellent (77.3)**	**NA**	**NR**	**77.5 (15.9)**
**HCSBS**	Merrell, Streeter [118]	2001^A^	-	-	-	-	-	-	-	Excellent (88.9)	NA
	Lund and Merrell [119]	2001^A^	-	-	-	-	-	Excellent (76.5)	-	-	NA
	Merrell and Caldarella [120]	1999^A^	Good (71.4)	-	-	-	Fair (41.7)	Good (70.8)	-	-	NA
	Merrell and Caldarella [156]	2000^M^	Excellent (85.9)	Good (74.2)	Excellent (86.3)	Good (57.1)	Good (75.0)	-	-	-	NA
	**TOTAL**	**NA**	**Excellent (78.7)**	**Good (74.2)**	**Excellent (86.3)**	**Good (57.1)**	**Good (58.3)**	**Good (73.6)**	**NR**	**Excellent (88.9)**	**73.9 (12.4)**
**IRS**	Anme, Shinohara [121]	2010^A^	-	-	-	-	-	Fair (29.4)	-	-	NA
	**TOTAL**	**NA**	**NR**	**NR**	**NR**	**NR**	**NR**	**Fair (29.4)**	**NA**	**NR**	**NA**
**IRS-Advanced**	Anme, Watanabe [122]	2011^A^	-	-	-	-	-	Poor (13.0)	-	-	NA
	Anme, Tokutake [123]	2014^A^	Fair (47.8)	-	-	-	-	Fair (34.8)	-	-	NA
	**TOTAL**	**NA**	**Fair (47.8)**	**NR**	**NR**	**NR**	**NR**	**Poor (23.9)**	**NA**	**NR**	**NA**
**IRS-BC TOTAL**	Anme, Sugisawa [124]	2012^A^	-	-	-	-	-	Fair (30.4)	-	-	NA
	**TOTAL**	**NA**	**NR**	**NR**	**NR**	**NR**	**NR**	**Fair (30.4)**	**NA**	**NR**	**31.9 (10.3)**
**MESSY-II** ^**b**^	Matson, Neal [125]	2010^A^	Good (57.3)	-	-	-	-	Good (60.9)	-	-	NA
	Matson, Neal [126]	2012^A^	Excellent (100)	-	-	-	Excellent (83.3)	-	-	-	NA
	Teodoro, Kappler [128]	2005^A^	Excellent (85.9)	-	-	-	Good (75.0)	Good (54.3)	Fair (39.3)	-	NA
	Méndez, Hidalgo [127]	2002^A^	Excellent (100)	-	-	-	Excellent (100)	Excellent (79.7)	Good (63.5)	-	NA
	Chou [129]	1997^A^	Good (57.3)	-	-	-	Fair (41.7)	-	Fair (30.4)	-	NA
	Matson, Horovitz [130]	2013^A^	Excellent (81.0)	-	Excellent (82.9)	-	-	-	-	-	NA
	**TOTAL**	**NA**	**Excellent (80.3)**	**Excellent (82.9)**	**NR**	**NR**	**Good (75.0)**	**Good (68.5)**	**Fair (44.4)**	**NR**	**70.2 (15.4)**
**PKBS-2** ^**b**^	Merrell [131]	2003^M^	Excellent (85.9)	Good (68.9)	Fair (48.4)	Excellent (78.6)	-	-	-	-	NA
	Merrell [133]	1995^A^	-	-	-	-	-	Good (73.2)	-	-	NA
	Canivez and Rains [134]	2002^A^	-	-	-	-	-	Excellent (82.6)	-	-	NA
	Canivez and Bordenkircher [135]	2002^A^	-	-	-	-	-	Excellent (82.6)	-	-	NA
	Carney and Merrell [136]	2002^A^	-	-	-	-	-	-	Fair (48.2)	-	NA
	Wang, Sandall [137]	2001^A^	Good (66.9)	-	-	-	-	Good (57.7)	-	-	NA
	Merrell [138]	1996^A^	-	-	-	-	Good (75.0)	-	-	-	NA
	Carney and Merrell [139]	2005^A^	-	-	-	-	-	Fair (47.8)	-	-	NA
	Edwards, Whiteside-Mansell [142]	2003^A^	Excellent (85.9)	-	-	-	Good (75.0)	-	-	-	NA
	Fernández, Benítez [140]	2010^A^	-	-	-	-	Good (75.0)	-	-	-	NA
	Al Awmleh and Woll [141]	2013^A^	Good (71.4)	-	Excellent (75.7)	-	-	-	Fair (36.4)	-	NA
	**TOTAL**	**NA**	**Excellent (77.5)**	**Good (68.9)**	**Good (62.1)**	**Excellent (78.6)**	**Good (75.0)**	**Good (68.8)**	**Fair (42.3)**	**NR**	**67.6 (12.6)**
**PSMS**	Fink, Rosnay [143]	2013^A^	Excellent (100)	Excellent (96.6)	-	-	Excellent (100)	Good (51.3)	-	Good (73.2)	NA
	**TOTAL**	**NA**	**Excellent (100)**	**Excellent (96.6)**	**NR**	**NR**	**Excellent (100)**	**Good (51.3)**	**NA**	**Good (73.2)**	**84.2 (21.6)**
**QRSH-PR**	Bolsoni-Silva, Marturano [144]	2009^A^	Excellent (90.5)	-	-	Excellent (85.7)	Excellent (83.3)	Good (62.0)	-	-	NA
	**TOTAL**	**NA**	**Excellent (90.5)**	**NR**	**NR**	**Excellent (85.7)**	**Excellent (83.3)**	**Good (62.0)**	**NA**	**NR**	**80.4 (12.6)**
**SCI**	Rydell, Hagekull [145]	1997^A^	Excellent (100)	Excellent (86.3)	-	-	Excellent (100)	Good (60.9)	-	-	NA
	**TOTAL**	**NA**	**Excellent100**	**Excellent (86.3)**	**NR**	**NR**	**Excellent (100)**	**Good (60.9)**	**NA**	**NR**	**86.8 (18.4)**
**SEARS-P**	Merrell, Felver-Gant [146]	2011^A^	Excellent (100)	Good (58.7)	-	Good (64.3)	Excellent (100)	Excellent (81.1)	-	-	NA
	Merrell [149]	2011^M^	Excellent (85.9)	Good (62.1)	-	Good (64.3)	Good (75.0)	Good (63.6)	-	-	NA
	**TOTAL**	**NA**	**Excellent (93.0)**	**Good (60.4)**	**NR**	**Good (64.3)**	**Excellent (87.5)**	**Good (72.3)**	**NA**	**NR**	**NA**
**SEARS-T**	Merrell, Cohn [147]	2011^A^	Excellent (85.9)	-	-	Fair (35.7)	Good (75.0)	Good (57.8)	-	-	NA
	Romer and Merrell [148]	2012^A^	-	Good (68.9)	-	-	-	-	-	-	NA
	Merrell [149]	2011^M^	Excellent (85.9)	Good (65.5)	-	Good (64.3)	Good (75.0)	Good (53.4)	-	-	NA
	**TOTAL**	**NA**	**Excellent (85.9)**	**Good (67.2)**	**NR**	**Fair (50.0)**	**Good (75.0)**	**Good (55.6)**	**NA**	**NR**	**NA**
**SEARS-C**	Romer and Merrell [148]	2012^A^	-	Good (72.3)	-	-	-	-	-	-	NA
	Merrell [149]	2011^M^	Excellent (85.9)	Good (62.1)	-	Good (64.3)	Good (75.0)	Fair (43.5)	-	-	NA
	**TOTAL**	**NA**	**Excellent (85.9)**	**Good (67.2)**	**NR**	**Good (64.3)**	**Good (75.0)**	**Fair (43.5)**	**NA**	**NR**	**NA**
**SEARS-A**	Romer and Merrell [148]	2012^A^	-	Good (72.3)	-	-	-	-	-	-	NA
	Merrell [149]	2011^M^	Excellent (85.9)	Good (62.1)	-	Good (64.3)	Good (75.0)	Fair (48.7)	-	-	NA
	**TOTAL**	**NA**	**Excellent (85.9)**	**Good (67.2)**	**NR**	**Good (64.3)**	**Good (75.0)**	**Good (56.4)**	**NA**	**NR**	**69.4 (14.2)**
**SEEC**	Sparrow, Balla [150]	1998^M^	Good (71.4)	Good (62.1)	Good (58.6)	Good (64.3)	Good (75.0)	-	-	-	NA
	**TOTAL**	**NA**	**Good (71.4)**	**Good (62.1)**	**Good (58.6)**	**Good (64.3)**	**Good (75.0)**	**NR**	**NA**	**NR**	**66.3 (6.8)**
**SP**	Donohue [151]	2005^A^	-	Excellent (92.8)	-	Excellent (100)	Good (58.3)	-	-	-	NA
	Donohue [152]	2007^A^	-	Good (72.3)	-	-	-	-	-	-	NA
	**TOTAL**		**NR**	**Excellent (82.6)**	**NR**	**Excellent (100)**	**Good (58.3)**	**NR**	**NA**	**NR**	**80.3 (20.9)**
**SSBS-2**	Merrell [17]	2002^M^	Excellent (85.7)	Excellent (93.1)	Good (65.5)	Excellent (100)	Excellent (100)	Excellent (78.3)	-	Excellent (80.0)	NA
	Raimundo, Carapito [153]	2012^A^	Excellent (90.5)	-	-	-	Excellent (83.3)	-	Good (60.6)	-	NA
	**TOTAL**	**NA**	**Excellent (88.1)**	**Excellent (93.1)**	**Good (65.5)**	**Excellent (100)**	**Excellent (91.7)**	**Excellent (78.3)**	**Good (60.6)**	**Excellent (80.0)**	**82.2 (13.8)**
**SSIS**	Gresham, Elliott [154]	2010^A^	-	Excellent (79.1)	-	-	-	Good (70.8)	-	-	NA
	Gresham and Elliott [155]	2008^M^	Excellent (87.4)	Excellent (79.1)	Excellent (86.3)	Excellent (85.7)	Fair (50.0)	Good (56.5)	Good (69.9)	-	NA
	**TOTAL**	**NA**	**Excellent (87.4)**	**Excellent (79.1)**	**Excellent (86.3)**	**Excellent (85.7)**	**Fair (50.0)**	**Good (63.7)**	**Good (69.9)**	**NR**	**74.6 (14.1)**
**-**	**OVERALL AVERAGE (Mean [SD])**	**NA**	**84.5 (12.9)**	**74.7 (11.9)**	**70.0 (12.8)**	**74.0 (17.0)**	**78.1 (14.8)**	**56.8 (17.5)**	**54.3 (13.2)**	**80.7 (7.9)**	NA

*Notes*. The measurement properties of each instrument were evaluated according to the COSMIN rating. Four-point scale was used (1 = Poor, 2 = Fair, 3 = Good, 4 = Excellent) and the outcome was presented as percentage of rating (Poor = 0–25.0%, Fair = 25.1%–50.0%, Good = 50.1%–75.0%, Excellent = 75.1%–100.0%)

^b^The psychometric properties of the previous version were examined from the studies published after 1994; BC = Between Children; NR: not reported; NA: not applicable.

## Discussion

In this systematic review, we identified and evaluated the quality of psychometric properties of instruments that measure social skills and behaviours developed after 1994. We identified 13 instruments that evaluated a component of social skills and behaviours that fit the definition we used in the review. The vast majority of instruments (11) were developed mainly for school aged children and adolescents, with two instruments solely developed for children 2–5 years (i.e., QRSH-PR and SEEC).

Alongside the validity evidence, reliability findings also need to be reported. This systematic review of social skills and behaviour instruments using the COSMIN framework provided a comprehensive summary of this. Application of the COSMIN checklist based taxonomy provided the framework for a critical evaluation of the quality and extent of psychometric evidence of the 45 research articles and manuals on the 13 social skills and behaviour instruments. Based on the COSMIN taxonomy, the social skills and behaviour instrument with the most robust psychometric properties to date was the SSBS-2, given that all eight psychometric properties were evaluated and it had an overall rating of excellent. Three measures (HCSBS [73.9%]; PKBS-2 [67.6%]; SSIS [74.6%]) evaluated seven of the eight psychometric properties; however, their overall ratings were good (not excellent). Five of the measures had overall excellent ratings, but have evaluated only five psychometric properties (ESI [77.5%]; PSMS [84.2%]), four psychometric properties (QRSH-PR [84.2%], SCI [86.8%], or three psychometric properties (SP [80.3%]) respectively. The IRS is the measure with the least evidence of having sound psychometric properties; having rated only two psychometric properties and achieving an overall fair rating (31.9%).

### Reliability and Validity

The COSMIN checklist provides information about the instruments' properties with reliability testing for internal consistency. While these aspects of reliability were not reported for a number of instruments, the current review showed good to excellent reliability for the majority of the instruments. However, only six of the thirteen instruments reported on measurement error. When selecting appropriate outcome measures for a study, consideration of the measurement error of the instruments is important as a small measurement error will allow the instrument to detect smaller treatment effects and allow for stronger conclusions to be drawn. Thus, clinical trials will require smaller sample sizes if the measurement error is small in relation to its minimal important change (MIC), compared with instruments where the opposite applies [159].

The results of the current systematic review revealed considerable variability and range of sample sizes used for the validation and development of measures. For instance, the ESI was developed and validate using a total sample size of 6,552 people classified under various diagnostic categories [117], whereas the IRS-BC [124] was validated with a sample of 20 children. Other measures which were developed and validated with small sample sizes included the IRS-BC [124], IRS-A [122, 123], SP [151, 152]. Large numbers in normative samples used for validation and development increases the generalisability of the results of measures to a population, and allows clinicians to make informed assessments about a client’s functioning in relation to a representative sample of people with similar characteristics (e.g., age, sex). In contrast, validation studies using a limited sample size are not considered adequate for reaching conclusions about the clinical findings of the measure, as the small number of participants does not allow for informed clinical assessment.

The theoretical construct being measured by an instrument must be clearly defined and then a body of evidence of the instrument’s construct validity must be accrued. Within the COSMIN taxonomy, construct validity is comprised of content validity, structural validity and hypothesis testing. Assessment of content validity revealed excellent quality for the PKBS-2, QRSH-PR, SP, SSBS-2 and SISS. However, the SCI, ESI, three versions of the IRS, MESSY-II and PSMS did not provide any evidence of content validity, highlighting a need for further research. Reported structural validity revealed that the SCI, ESI, MESSY-II, four versions of the SEARS, PSMS, PKSB-2, SSBS-2 and SEEC all had published evidence of their structural validity leading to rankings of ‘excellent’. Conversely, the three versions of the IRS did not have any published information in this domain; again highlighting a need for further research. The majority of the social skills and behaviour instruments (11 of 13) provided evidence of hypothesis testing ranging at the ‘good’ to ‘excellent’ level. Only the SP and the SEEC did not provide any evidence of hypothesis testing. Evidence for both cross-cultural validity (four measures: SSIS, MESSY-II, PKBS-2, and SSBS-2) and criterion validity (three measures: HCSBS, PSMS, and SSBS-2.) were the least reported psychometric property of validity.

When a scale is used without the documented measurement properties (such as construct validity) it can have potentially negative consequences, such as an error in clinical judgment or practitioners inaccurately interpreting assessment findings. Being able to investigate how well a scale measures what it claims to measure and its ability to hold its meaning across varied contexts and sample groups is vital so that it can be used with confidence in clinical settings. This systematic review of social skills and behaviour instruments provides a concise summary of the current state of play of the psychometric properties of these scales.

The importance of external or environmental influences has been emphasised by numerous theorists and researchers; however, few studies have examined the psychometric properties of an instrument in the different environmental contexts within which the social interaction occurs. It is likely that the environment (family, organisational or institutional structures, community, education, and culture) has a substantial impact on the social functioning of children and youth with and without disabilities. Therefore, future investigations of cross-cultural validity would seem valuable in the development of instruments that purport to measure social skills and behaviours.

### Definition of Social Functioning as a Construct

In line with discrepancies within the theoretical models and frameworks [1, 19], there was only moderate consensus between the instruments when the stated purposes of all instruments were compared. Among the stated aims of the instruments, the stated purpose was that they measured social competence, child-child interactions, social behaviours, or social and emotional problems, adjustment, or functioning. All terms were in agreement with widely accepted and long standing definitions of the components of social functioning as involving internal or person-related factors (i.e., cognitive, affective, linguistic and personality traits) [57, 160]. However, it remains problematic that a uniform overarching definition of social functioning remains elusive within a body of research that focuses on the reliable, valid, and responsive measurement of social functioning.

This is particularly so when current conceptual models highlight the influence of external factors as well [1]. The purpose of an instrument needs to include a robust definition or statement about the construct(s) that an instrument seeks to measure [46]. In the 13 instruments evaluated in this review, the articulated constructs may be viewed as the instrument developers’ attempt to operationalise an important aspect of social functioning. Before evaluating the merits and weaknesses of the published data about multiple social interaction assessments, the first step is to situate the constructs they measure within theoretical underpinnings. This is necessary whether the instrument measures a single entity (e.g., expressive language) or part of an overarching broader construct (e.g., social functioning). Accordingly, we recommend communication and open discourse among researchers and practitioners who strive to operationalise social functioning as a universally acceptable and defined construct. In the absence of a universally agreed framework, the overlapping yet unclear differentiation of social functioning constructs, collectively described, may prove confusing for both researchers and practitioners. One way to overcome the heterogeneity of definitions is to apply a universally accepted framework with clear interrelated concepts. The International Classification of Functioning [161] has the potential to lend itself to be such a framework.

### Application of the International Classification of Functioning (ICF)

The World Health Organization promotes the ICF as a potential guiding framework for professionals, organisations and governments seeking to address social and health inequalities among people with disabilities [161]. The ICF is non-discipline specific, theoretically neutral, and is based on the social model of disability. The ICF offers the advantage of being compatible with current leading psychological models and theorists described previously [1, 16]. Such theories recognise internal factors such as brain integrity and personality (ICF: body structure and function and person factors), external factors such as family, organisational/institutional places (ICF: environmental factors) and engagement and participation within the person’s natural environments (ICF: activity participation). Furthermore, evidence of cross-cultural validity testing would provide evidence of the relationships between ICF person and environment factors. Social skills and behaviour, as widely defined in this review, encompasses several key aspects of the ICF including a description of body structure and function (i.e. voice and speech functions); activity and participation factors (learning and applying knowledge, communication, domestic life, interpersonal relations and interactions, and community, social and civic life); person factors (i.e. age, gender, education level, culture); and environmental factors (support and relationships, attitudes, services, systems, and policies).

### Limitations

This systematic review has a number of limitations. Information published in languages other than English were not included; therefore, some research findings may have been overlooked. Not all authors who published research on the psychometric properties of social skills and behaviour instruments were directly contacted; therefore, information may have been neglected. Evaluating the quality of responsiveness as a psychometric property was outside the scope of this systematic review, due to the size of this systematic review. We are of the opinion that evaluating the responsiveness of the included instruments warrants a review in itself, given that the number of papers to be evaluated would increase exponentially. Instruments developed for specific clinical populations were outside the scope of this systematic review and they may have sound psychometric qualities for clinical use; further research is needed to evaluate this.

### Implications for Practice and Future Research

A number of implications arise from the findings of this systematic review. Measuring social functioning is complex as it involves numerous distinct yet related social skills that are used during interactions with multiple interactants with varying levels of social competence within a multitude of contexts. Therefore it is unlikely that one measure can address all the assessment needs of researchers and practitioners. As such it is not prudent to recommend one singe measure for use. The SSBS-2 is the social skills and behaviour measure with the most robust psychometric properties. Of particular strength is that all eight psychometric properties have been investigated. The SSBS-2 is recommended for use as a screening measure in educational settings. The PKBS-2 also has sound psychometric properties and is recommended for use as a diagnostic measure of social and emotional problems in children with significant behavioral, emotional and developmental problems. The SSIS has sound psychometric properties, but was clearly developed as an outcome measure; thus needing it to detect change over time following an intervention. Responsiveness of the SSIS is therefore one of the most important psychometric properties. As the review did not evaluate responsiveness, it is not possible to make a recommendation for the purpose it was developed. The HCSBS is another measure with robust psychometric properties and is recommended as a screening measure for home and community contexts. While there are other measures that were appraised in this review that show great promise in terms of the available evidence of the quality of their psychometric properties, more research is needed to evaluate the psychometric properties that have not been reported on to date.

It is important that researchers and practitioners utilise instruments with sound psychometric properties in support of evidence-based and research practices. It is recommended that practitioners collaborate with researchers to further develop the body of knowledge related to the reliability and validity of the social skills and behaviour scales. There is a need for ongoing research in the area of the psychometric properties of social skills and behaviours instruments. The body of psychometric evidence for instruments is dynamic and constantly being added to. It is strongly recommended that a universal working definition of social functioning as an overarching construct plus any related sub-constructs be generated. This would ensure that a consistent approach to evaluating the outcomes of social skills interventions is followed. In particular, it is recommended that the cross-cultural validity and criterion validity of all 13 instruments be further investigated.

There is a need to evaluate the responsiveness of the instruments and therefore to evaluate their suitability for use as an outcome measure of social skills and behaviour. Measures can be prognostically used to report on the responsiveness to a particular intervention or analytically to detect within subject change or between subgroup differences [45]. In an evidence-based practice era for all professionals who may work with clients presenting with impaired social functioning, the development of appropriate and psychometrically sound measurements is crucial to substantiate the effectiveness of interventions and programs. Consequently, instruments require statistical evaluation to determine stability over time in the absence of an intervention, as well as reliability, prior to thorough investigations of responsiveness and sensitivity to change over time. Of the included measures, a considerable number of measures had been validated within the last 10 years. Only the SEEC [150] and SCI [145] had not re-evaluated or updated their psychometric properties within the last 10 years. Furthermore, the SEEC and SCI measures had only been evaluated on a singular occasion. Future evaluation of psychometric properties is needed to determine the stability of these measures over time.

## Conclusion

This systematic review presented the results of 45 studies and manuals that reported evidence of the psychometric properties of 13 social skills and behaviour instruments used with children and youth. The COSMIN taxonomy was used to rate the reliability and validity information reported about the instruments. Three social skills and behaviour scales were found to have the strongest level of psychometric evidence reported in at least seven of the eight psychometric properties that were appraised. The authors recommend that practitioners and researchers consider using the robust SSBS-2, HCSBS and PKBS-2 with children and youth for the purposes and context for which they have been developed. It is also recommended that a more consistent definition of social skills and behaviours as a construct be generated. Only the SSBS-2 has reported on all the psychometric properties evaluated in this systematic review. There is a need for the authors of the measures included in this systematic review to evaluate and report on the quality of the psychometric properties that have not been assessed to date. The body of psychometric evidence of any scale or measure is constantly changing and evolving and it is important for practitioners to be knowledgeable of the best instruments and outcome measures for use when monitoring and assessing children’s social functioning.

## Supporting Information

S1 Table PRISMA 2009 Checklist. From: Moher D, Liberati A, Tetzlaff J, Altman DG, The PRISMA Group (2009). Preferred Reporting Items for Systematic Reviews and Meta-Analyses: The PRISMA Statement. PLoS Med 6(6): e1000097. doi:10.1371/journal.pmed1000097.
(PDF)
